# Disutility associated with cancer screening programs: A systematic review

**DOI:** 10.1371/journal.pone.0220148

**Published:** 2019-07-24

**Authors:** Lin Li, J. L. (Hans) Severens, Olena Mandrik

**Affiliations:** 1 School of Health Policy & Management, Erasmus University Rotterdam, Rotterdam, The Netherlands; 2 Institute for Medical Technology Assessment (iMTA), Erasmus University Rotterdam, Rotterdam, The Netherlands; 3 The University of Sheffield, School of Health and Related Research (ScHARR), Health Economic and Decision Science (HEDS), Sheffield, United Kingdom; University of Turin, ITALY

## Abstract

**Objectives:**

Disutility allows to identify how much population values intervention-related harms contributing to knowledge on the benefits/harms ratio of cancer screening programs. This systematic review evaluates disutility related to cancer screening applying a utility theory framework.

**Methods:**

Using a predefined protocol, Embase, Medline Ovid, Web of Science, Cochrane, Google scholar and supplementary sources were systematically searched. The framework grouped disutilities associated with breast, cervical, lung, colorectal, and prostate cancer screening programs into the screening, diagnostic work up, and treatment phases. We assessed the quality of included studies according to the relevance to target population, risk of bias, appropriateness of measure and the time frame.

**Results:**

Out of 2840 hits, we included 38 studies, of which 27 measured (and others estimated) disutilities. Around 70% of studies had medium to high-level quality. Measured disutilities and Quality Adjusted Life Years loss were 0–0.03 and 0–0.0013 respectively in screening phases. Both disutilities and Quality Adjusted Life Years loss had similar ranges in diagnostic work up (0–0.26), and treatment (0.09–0.27) phases. We found no measured disutilities available for lung cancer screening and—little evidence for disutilities in treatment phase. Almost 40% of the estimated disutility values were above the range of measured ones.

**Conclusions:**

Cancer screening programs led to low disutities related to screening phase, and low to moderate disutilities related to diagnostic work up and treatment phases. These disutility values varied by the measurement instrument applied, and were higher in studies with lower quality. The estimated disutility values comparing to the measured ones tended to overestimate the harms.

## Introduction

Cancer is one of the most wide-spread chronic diseases with an estimated 18.1 million of new cases in 2018 leading to 9.6 million deaths worldwide.[1] Among all malignancies, the diseases with the highest five-year prevalence in 2017 were breast (19%), prostate (12%), colorectal (11%), lung (5.8%), and cervical (4.8%) cancers. [2]

Cancer screening programs can help to detect the disease before symptoms appear. Empirical studies show the benefits of cancer screening in decreasing the mortality of the most prevalent cancers. For example, the US Preventive Services Task Force meta-analyses showed 15%- 20% reduction in breast cancer mortality with mammography screening and 20–60% reduction in cervical cancer mortality with cytology-based screening. [3,4] The International Agency for Research on Cancer reported 18–31% reduction of colorectal cancer mortality due to sigmoidoscopy screening, [5] while the National Cancer Institute reported a 20% reduction in lung cancer mortality among smokers with low dose computed tomography screening.[6]

While some of the cancer screening programs (for breast, cervical, and colon cancers) are widely implemented, there are increasing concerns on possible harms of screening.[5,7] These harms mainly include anxiety, procedural risks, false positive diagnosis, and overdiagnosis (diagnosing cancers that would never have caused any symptoms).[8–11] Prevention strategies must be first of all safe, and so governmental bodies pay high attention to assessments of possible screening-related harms which could lead to retreat or delay of cancer screening programs. [12–14]

Harms can be either assessed from clinical endpoints or represented by patients’ values for the outcomes. Preferences of population for screening programs may be expressed in utility values, while screening-related harms (or loss in health-related quality of life) illustrated in disutility values. [15] Methods of deriving health state utility values (HSUVs) include direct and indirect methods. The examples of direct approaches include Time Trade Off (TTO), Standard Gamble (SG), Visual Analog Scale (VAS), and Discrete Choice Experiment (DCE). Among the indirect instruments are EuroQol 5 Dimensions (EQ-5D), Short Form 6 Dimension (SF-6D), Rand-36, and Health Utilities Index (HUI). [16] These methods are rooted in utility theories that reflect the consumer satisfaction over the choices. [17,18]

Utilities as measures of patient preferences are widely considered in health decision making, as a component of quality adjusted life years (QALYs) in cost-effectiveness analysis. The QALYs are calculated as HSUVs multiplied by time spent in certain health state (called time frame). Theoretically the disutility value equals to “1-utility”, so the larger disutility related to screening then the lower total utility for the screened population. QALYs losses (disutility value multiplied by time frame) express the general harms of the screening program.

Knowledge on screening-related disutilities is a crucial component in understanding of benefits/harms ratio of cancer screenings. Meanwhile, no systematic review summarized this evidence so far. [19–21] Our study aims to fill in this gap by identifying typologies of disutilities and further evaluating the reliability and variability in disutility values.

## Methods

### Search and selection

We systematically searched Embase, Medline Ovid, Web of Science, Cochrane, and Google scholar from their commencements till April 2018. The search syntax (S1 File) was developed with an input from a qualified librarian. We also searched non-systematically the other supplementary sources (S2 File) and references of the included studies.

One researcher (LL) screened and included all abstracts focused on lung, breast, colorectal, cervical or prostate cancers reporting the results of studies of various designs (models, randomized controlled trials, cohort or case-controlled studies, and systematic reviews). We excluded studies that were: (1) related to other diseases; (2) reporting clinical utility/practice (for example, screening methodology, compliance, clinical diagnosis or treatment); or (3) not full-text papers (meeting proceedings, posters or commentaries). All full texts of included abstracts were double screened (by LL and OM) excluding studies that did not report disutility values or reported disutility values cited from another source (in this case the original source was used). If the author used disutility from the literature but also applied certain assumptions for the value, and therefore its value differs from the cited value, then it was included as estimated value.

All the relevant information from the included studies was extracted by one author (LL) using a data extraction form, and verified by the second author (OM).

### Theoretical framework of the review

**Fig 1:**
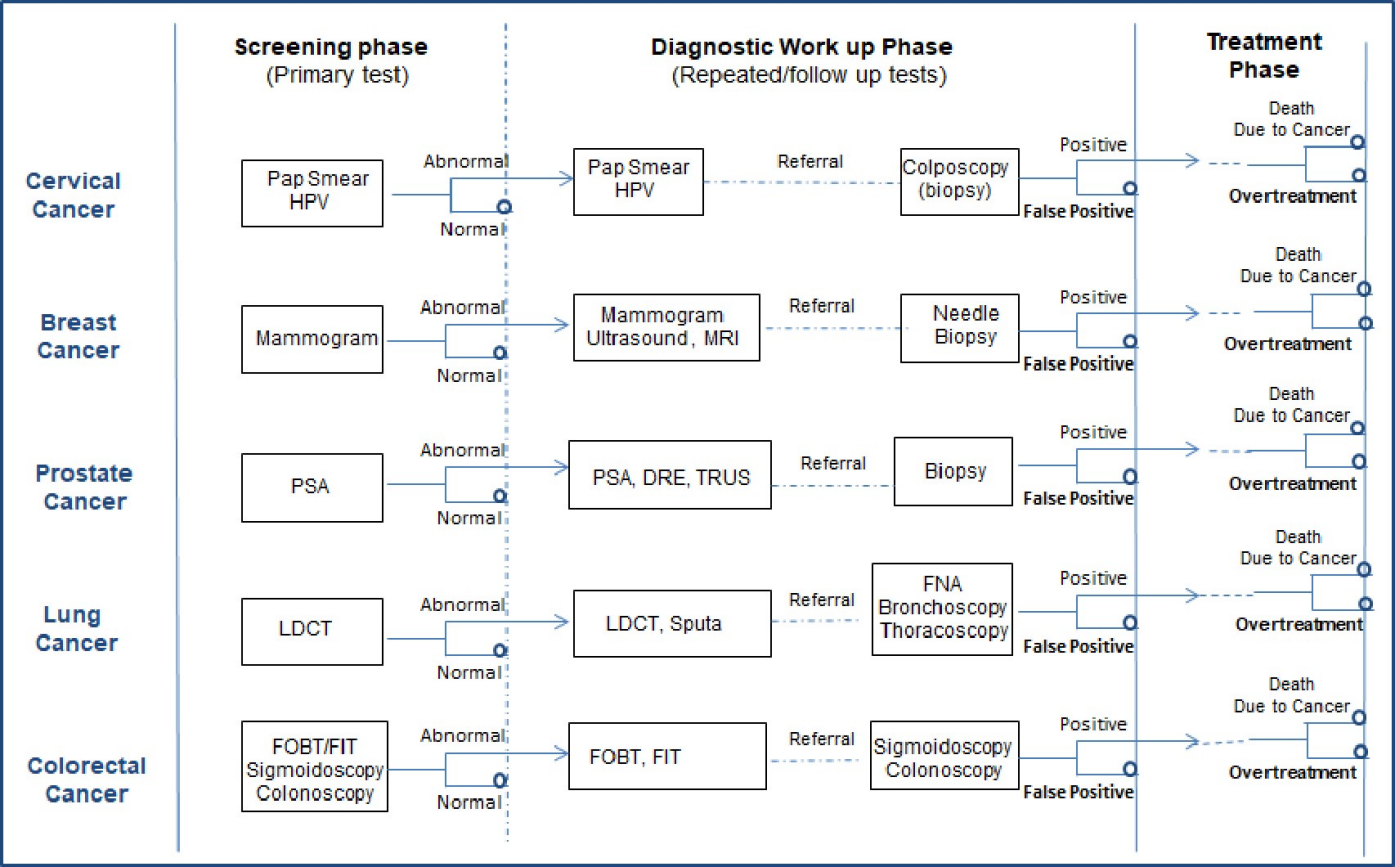
Disutility typologies. Notes: HPV = Human papillomavirus; PSA = prostate specific antigen; MRI = Magnetic resonance imaging; DRE = digital rectal examination; TRUS = trans-rectal ultrasound; LDCT = Low dose Computed Tomography; FNA = Fine needle aspiration; FOBT = fecal occult blood testing; FIT = fecal immunofluorenscence testing.

Referring to the American College of Physicians’ value framework for cancer screening,[12] we grouped the reported disutility into three typologies (Fig 1).

- Screening phase: the disutility is normally derived from the primary screening test because of the discomfort during the procedure and have a short-term effect, generally from a few days and up to 3 weeks.
- Diagnostic work up phase: the disutility at this stage is not only caused by physical effects (such as discomfort or complication from follow up or repeated tests’ procedure), but also by psychological effects (such as anxiety and emotional distress about unfavorable or indeterminate result). The time frame of this stage ranges from a few weeks to a few months.
  Disunities in this phase were divided into three groups:
  ◆ False positive results
  ◆ Procedure-wise
  ◆ Abnormal results.
- Treatment phase: The disutility in this phase is related to overtreatment of the screening detected (or overdiagnosed) cancer. The time frame generally ranges from several months to years.

### Quality appraisal

We developed the quality appraisal criteria based on Ara et al. (2017) [22] and Papaioannou et al (2013) [23] (Table 1). For each of the criteria the studies were scored as ‘good’ (score 2), ‘fair’ (score 1) or ‘poor’ (score 0). The studies with an overall score of ≥7, 5–6, 3–4, < 3 were rated as high, medium, low, very low quality respectively. Quality of the included studies was assessed independently by two reviewers (LL, OM) with disagreements being solved by consensus.

**Table 1:** Checklist for quality appraisal.

Criteria	Description
**Relevance to the population's preference**	
Respondent selection and recruitment	Does this result in a population comparable to that being evaluated?
Inclusion/exclusion criteria	Do the criteria exclude any individuals? (for example, the elderly >80-year-old are often not included in studies)
Relevance of location	Are the population recruited from multiple locations?
**Quality assessment—Risk of bias**	
Sample size	Is the sample size appropriate in reflection population’s preference?
Response rates to the measure used	Are the response rates reported? If so, are the rates likely to be a threat to the validity of the estimated health state utility values?
Loss to follow-up	How large is the loss to follow-up and are the reasons for it given? Are these likely to threaten the validity of the estimates?
Missing data	Are missing values well-reported and addressed? What are the levels of missing data and how are they dealt with? Could this threaten the validity of the estimates?
**Appropriateness of measure of disutility values**	
Appropriate use of instrument	For direct methods (DCE, TTO, SG, VAS): Is the method used appropriately? If anchors are used describing the perfect and worse health (for example anchored at 1 as equivalent to full health and 0 as equivalent to dead)?
	For indirect method (EQ-5D, SF-6D, SF-36, HUI): Are the adequate details of the method provided (for example, the details given on the version used, the social tariff applied, etc.)?
**Time frame**	Is the time frame specified? If so, is it sufficient or reliable to account for the magnitude of harm from screening (when relevant)? Time frame preferences: Measurement > guideline recommendation or assumption with justification (example, referring to a local clinical practice, or using the time frame from literature reviews) > assumption without justifications or no time frame reported (this criterion was considered as not applicable for DCE studies)

Notes: DCE = Discrete Choice Experiment; TTO = Time Trade Off; SG = Standard Gamble; VAS = Visual Analog Scale; EQ-5D = EuroQol 5 Dimensions; SF-6D = Short Form 6 Dimension; SF-36 = Short From 36; HUI = Health Utilities Index

### Data synthesis

We reported the disutility values by typology and cancer type respectively. The study aimed to combine the disutility values in meta-analysis under conditions of sufficient number of values (at least ten studies to each covariate [22]) and manageable heterogeneity in methods and outcomes. Considering that these conditions could not be reached, the qualitative synthesis was applied. We summarized mean and confidence interval disutility values by typology and cancer types; for studies of high and medium quality, we calculated non-reported confidence intervals when standard deviation was available.

## Results

### Studies selection and overview

**Fig 2:**
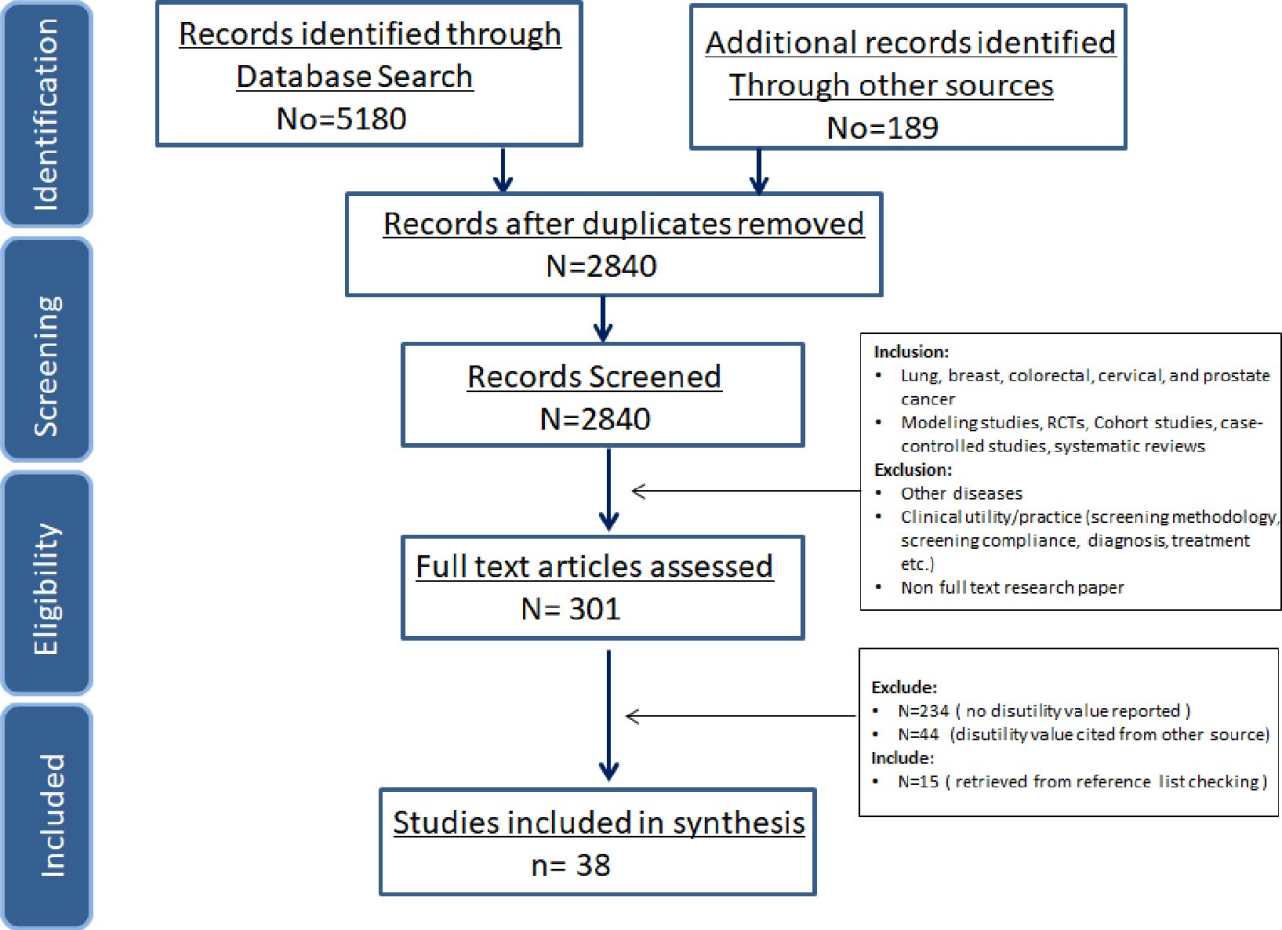
PRISMA flow chart of study selection process.

Out of 2840 abstracts identified by databases search and from the other sources, 23 studies met the eligibility criteria set by this review. Through checking the reference list of the articles, another 15 studies were included, resulting to 38 papers included in total (Fig 2). The level of agreement between two reviewers was high (kappa coefficient = 0.99).

A summary of characteristics of the included studies is provided in the Table 2. About 30% of the studies reported estimated disutility values, while remaining 27 studies evaluated disutilites by direct or indirect instruments. The data extraction form reporting key characteristics of individual studies is presented in the S1 and S2 Tables.

**Table 2:** Overview of the included studies (n = 38).

**Study Design**		
Modeling study		N = 15
Randomized controlled trial		N = 5
Observational study		N = 18
**Cancer Type**		
Colorectal cancer		N = 6
Cervical cancer		N = 17
Breast cancer		N = 10
Lung cancer		N = 2
Prostate cancer		N = 4
**Disutility Typology**		
Screening phase N = 15		
Diagnostic work up phase N = 34		
Treatment phase N = 3		
**Instruments used for measurement**		
Estimation		N = 11
Direct method		N = 21
	TTO	N = 4
	SG	N = 5
	VAS / RS	N = 9
	DCE	N = 3
Indirect method		N = 15
	EQ-5D	N = 8
	SF-6D	N = 3
	RAND / SF-36	N = 3
	HUI	N = 1
**Respondent**		
	Average-risk population	N = 22
	High-risk population	N = 2
	Healthcare professional/expert	N = 4
**Time frame**		
	Measurement	N = 10
	Guideline	N = 4
	Assumption	N = 19

**Fig 3:**
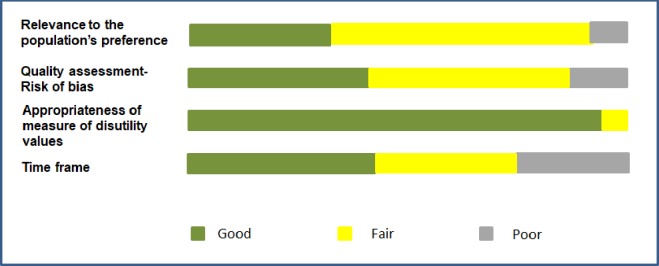
Overview of quality appraisal result per individual criteria.

Around 70% of studies which measured disutility values were rated as medium or high level of quality (S3 Table). The studies were ranked lower on risk of bias and time frame than the other quality criteria (Fig 3).

### Result on measured disutility

#### Disutility in screening phase

**Fig 4:**
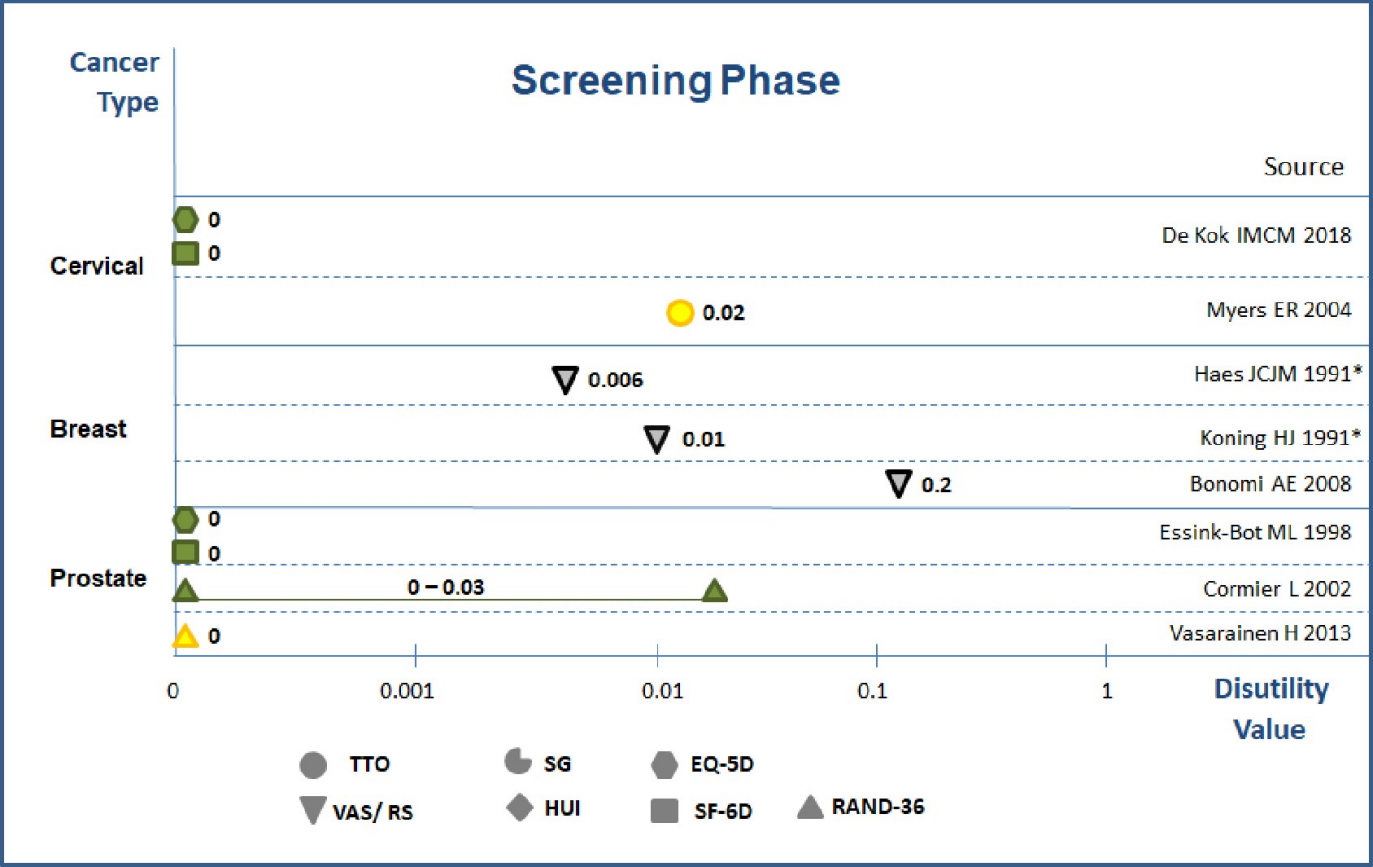
Disutility values in screening phase. Notes:1) Green color = high quality; Yellow color = medium quality; Grey color = low quality 2) with * values from experts; without * form general population.

Eight studies reported disutility related to cervical, breast and prostate cancer screenings (Fig 4). The disutility associated with cervical cancer screening ranged 0–0.02 [24,25], and QALY lost 0–0.0006 with 1 to 2 week timeframe. Disutilities related to breast cancer screening measured with VAS varied–considerably (0.006–0.2).[26–28] Disutility related to prostate cancer screening ranged 0–0.03 and calculated maximum QALYs loss around 0.0013, with most studies concluding on no disutility from screening attendance. [29–31] In a nutshell, taken the evidence from medium to high quality studies, the disutility values due to primary screening attendance were around 0–0.03, and the corresponding QALYs loss around 0–0.0013.

#### Disutility in diagnostic work up phase

**Fig 5:**
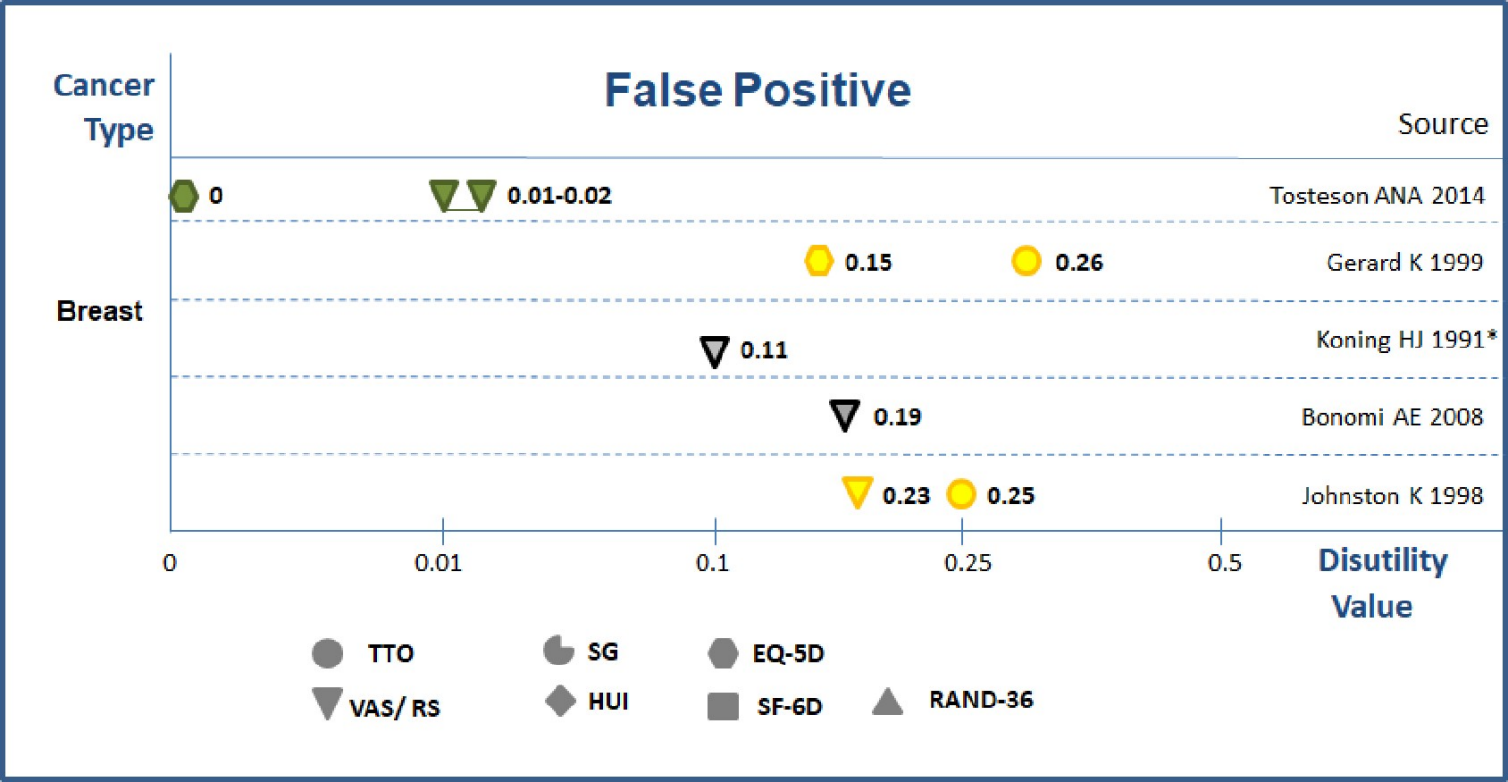
Disutility values for false positive in diagnostic work up phase. Notes: 1) Green color = high quality; Yellow color = medium quality; Grey color = low quality 2) with * values from experts; without * form general population.

**False positive.** Five studies assessed disutility of false positive result in breast cancer screening [27, 28, 32–34] (Fig 5) in the range of 0–0.26. Taken into account of the reported time frames (near 12 months), the calculated QALYs loss were around 0–0.26. Gyrd-Hansen *et al* (2001) used the DCE method to investigate preferences to cancer screening programs; the authors concluded that false positive diagnosis has no impact on utility values for colorectal cancer screening while marginal disutility was confirmed due to false positive result in breast cancer screening. [35] In summary, evidence from above studies showed that the false positive’s disutility values and calculated QALYs loss were in the range of 0–0.26.

**Fig 6:**
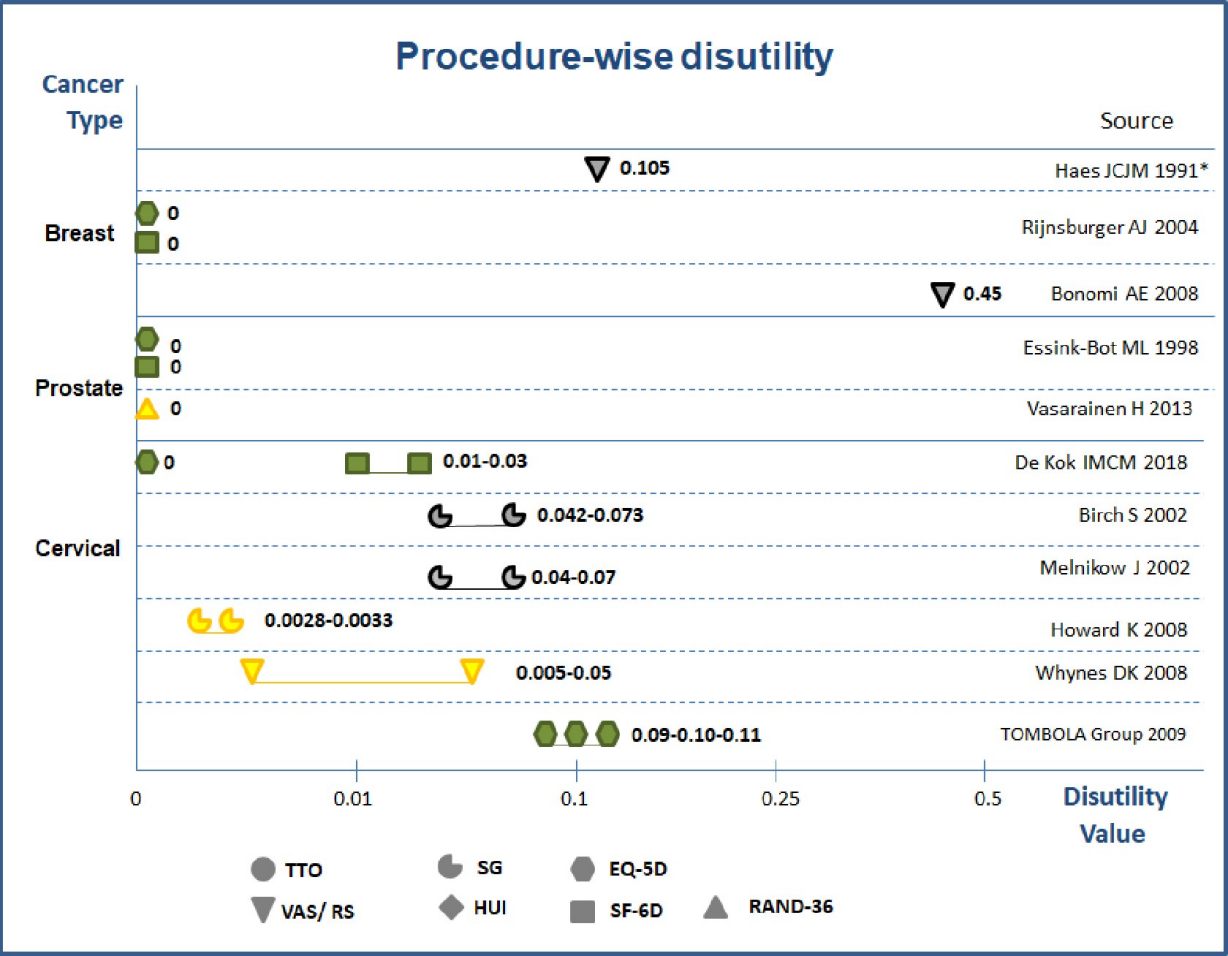
Procedure-wise disutility values in diagnostic work up phase. Notes: 1) Green color = high quality; Yellow color = medium quality; Grey color = low quality 2) with * values from experts; without * form general population 3) Different protocol: Conservative process includes observation, surveillance, follow-up with pap smear tests, aggressive process includes early colposcopy, immediate HPV tests.

**Procedure-wise disutilities.** Eleven studies reported the measured disutility values due to screening procedures for breast, prostate and cervical cancers (Fig 6).

Three studies on breast cancer reported procedure-wise disutilities in the range 0–0.45. [26, 28, 36] No disurilitues found for prostate cancer because of screening procedure. [29, 31] For cervical cancer, one study tested the procedure-wise disutility related to the repeated pap smear and colposcopy referral; the disutility values ranged 0–0.03 and the calculated QALYs loss 0–0.0375. [24] Another five studies investigated the differences in disutility of aggressive versus conservation protocols for patients with abnormal primary cervical cancer screening results. [37–41] The conclusions were contradictory whether early colposcopy leads to loss [38, 39] or gain in utilities.[40] Two studies concluded on disutilities of either immediate human papilloma virus (HPV) test [37] or immediate treatment and cytological surveillance versus conservative protocols.[41] Two DCE studies in colorectal cancer screening demonstrated the disutility of unnecessary colonoscopy and non-accurate or low-sensitivity tests from general population perspectives, [42, 43] while Marshell et al (2009) found no disutility of related to colonoscopy usage from physician’s preferences.[43] In general, leveraging the evidence from medium to high quality studies, the procedure wise disutility were 0–0.03, and the overall QALYs losses were in the range of 0–0.0375.

**Fig 7:**
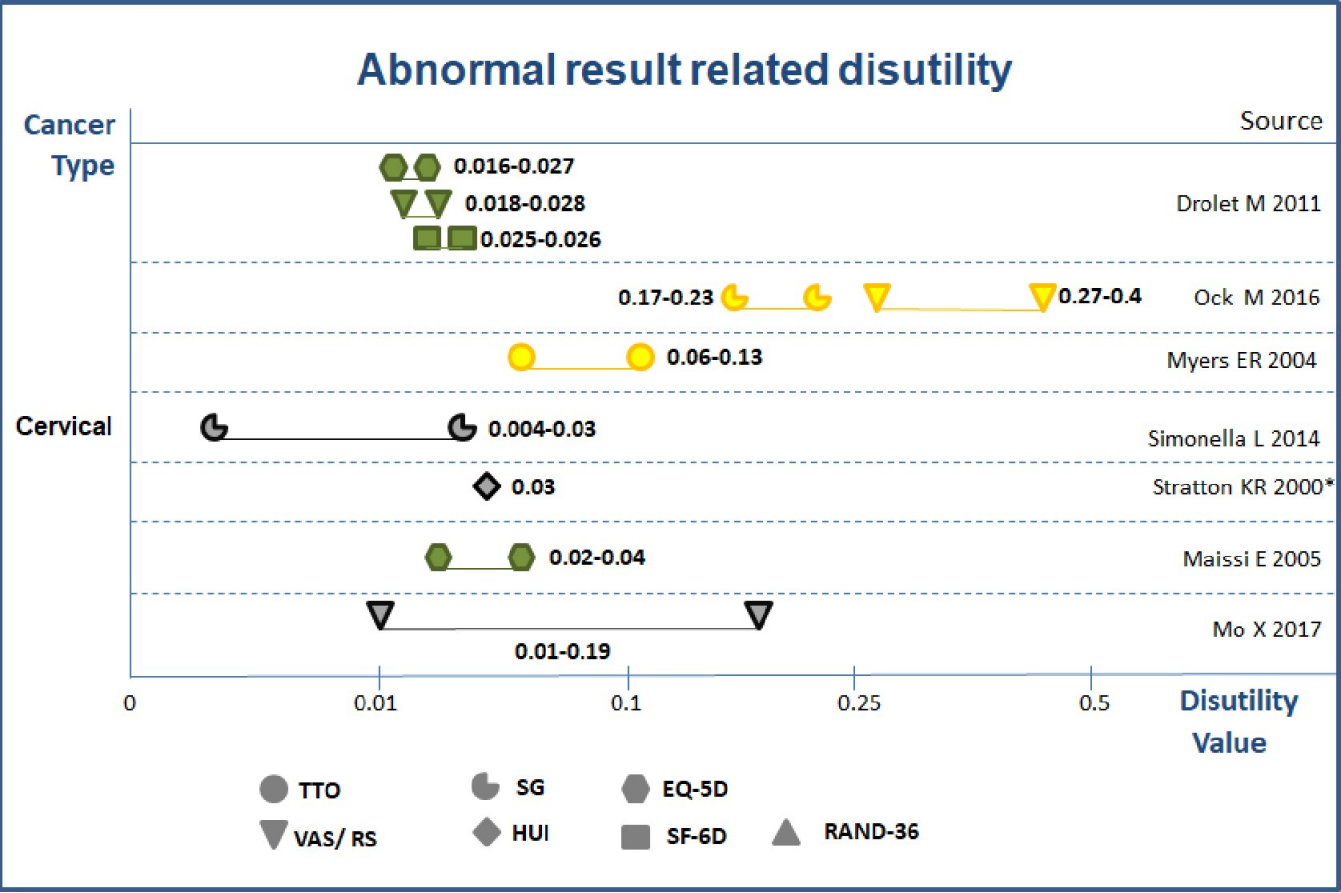
Abnormal result related disutility values in diagnostic work up phase. Notes: 1) abnormal result includes borderline or mildly dyskaryotic (BMD), cervical dysplasia, atypical squamous cells of undetermined significance (ASCUS), low-graded squamous intraepithelial lesion (LSIL), high—graded squamous intraepithelial lesion (HSIL), cervical intraepithelial neoplasia (CIN), Human papillomavirus positive 2) Green color = high quality; Yellow color = medium quality; Grey color = low quality; Dark color = very low quality 3) with * values from experts; without * form general population.

**Abnormal result related disutility.** Seven studies reported substantial variability in disutility values (the lowest of 0.004 for HPV positive and the highest of 0.4 for cervical intraepithelial neoplasia [CIN] II-III) and time frames (from 3 to 18 months) because of abnormal results. (Fig 7) [25, 44–49]

#### Disutility in treatment phase

The only study reporting disutility in treatment phase, by Cantor S.B. et al (2008) investigated the couples’ preference for prostate cancer screening outcomes. Disutility values of 0.09–0.27 were reported because of possible side effects (such as impotence, urinary incontinence, and injury) from the screening and consequent treatments. [50]

#### Summary on measured disutilities by cancer types

Most included studies were on cervical and breast cancers, while no single study reported the disutility of lung cancer screening. Similarly, only one study assessed disutility related to treatment phase—overtreatment of prostate cancer (Table 3).

**Table 3:** Summary on measured disutility studies by typology and cancer types.

Studies Number	Screening Phase	Diagnostic work up Phase			Treatment Phase
		False Positive	Procedure wise Disutility	Abnormal result related Disutility	Overtreatment
**Colorectal cancer**		1	2		
**Cervical Cancer**	2		6	7	
**Breast Cancer**	3	5	3		
**Lung Cancer**					
**Prostate Cancer**	3		2		1

#### Disutility values by studies’ quality and instrument used

Disutility values varied by quality of the studies, with values from high-quality studies being generally lower than from medium and low quality studies (Figs 4–6). At the same time, elicitated disutility values from indirect method were lower than those values from direct methods in screening-phase, false positive and procedure-wised disutility (Figs 4–6).

#### Result on estimated disutility

Out of eleven studies that reported the estimated disutility values, almost 40% assumed values outside of the measured range (Table 4).

**Table 4:** Overview of the estimated disutility values.

Typology/Cancer type	Range of measured disutility values by instrument	Range of estimated disutility values	If estimation is within the range of measured disutility value (Yes or No)	Reference
**Screening phase**				
colorectal cancer	NA	0.5–1.0	NA	[51–53]
cervical cancer	0–0.02	0.006	Yes	[54,55]
breast cancer	0.006–0.2	0–0.75	No	[56,57]
**Diagnostic work up phase**				
**a) False positive result**				
cervical cancer	NA	0.005–0.04	NA	[54, 55,58]
breast cancer	0–0.26	0.75	No	[57]
lung cancer	NA	0.02–0.6	NA	[59,60]
**b) Procedure wise**				
breast cancer	0–0.45	0.158	Yes	[56]
lung cancer	NA	0.1–0.5	NA	[60]
**c) Abnormal result**				
cervical cancer (CIN only)	0.01–0.40	0.03–0.12	Yes	[54,55,58,61]
**Treatment phase**				
colorectal cancer	NA	0.05–0.70	NA	[51, 59]

Notes: NA = not available CIN = cervical intraepithelial neoplasia

## Discussion

Our systematic review identified screening, diagnostic work up and treatment phases as three typologies of disutilities in cancer screening programs. Among these typologies the diagnostic work-up phase and treatment phases are potentially more important taking into account the impact on quality of life in terms of a degree of perceived screening–related harms and its time frame. Considering the analyzed literature on cervical, breast, and prostate cancer, we assume a low level of harms (less than 0.03 resulting to 0–0.0013 QALY loss) related to disutility from primary screening and low to moderate level of harms (0–0.26 range for both disutility and QALY loss) related to diagnostic work up from population perspective. Although women with false positive diagnosis considered the risk of having it as acceptable,[62] disutilities and QALYs loss related to false positive rate should not be ignored because of its commonality in clinical practice (for example, 1–11% in screening mammography [63–65]). Although this review identified only one study reporting disutility for treatment phase (0.09–0.27), taken into account the longer timeframe for disutilities related to overdiagnosis, we assume a moderate level of harms perceived in treatment phase.

Our review identified the studies both measuring and estimating disutilities related to cancer screening. An important finding of our review is that when disutilities are based on assumption, investigators tend to overestimate the harms; these methodological risks should be considered in cost-effectiveness analyses of cancer screening interventions.

Another important outcome of our review is application a novel framework to assess quality of studies reporting utility values. Quality of studies on HSUVs in cancer realm is rarely evaluated and so methodological improvements on this regard are important. One of the few evaluable estimates is a systematic review of Carter et al (2015), who qualitatively evaluated the quality of upper digestive tract cancer studies. [66] In our review, about 70% of studies on five cancers were ranked as medium / high quality. Meanwhile, the studies had important limitation in reporting uncertainty in their findings with only two of them including confidence intervals. For seven studies it was possible to derive the confidence intervals based on the data reported. While this information did not change the conclusions of the review, it undervalues even more the importance of disutilities in screening and diagnostic work up phase, with negative confidence intervals received in three high and medium quality studies.

An interesting observation, is that the higher quality the studies was rated, the lower disutility values were reported.

### Methodological considerations on disutility measurement

Variations in utility elicitation are strongly associated with the instruments used in the study. [15, 19, 67] We found that the indirect methods tended to retrieve lower disutility value than the direct methods, in most cases showing no at all. The published literature reported that utility values were generally higher with TTO than with SG, and generally lower with VAS/Rating Scale (RS). [68–70] In our review due to limited values retrieved, the head-to-head comparison among TTO, SG and VAS was not feasible. Meanwhile, we observed a trend of higher disutility values from TTO than VAS/RS. Considering stated, synthesis and interpretation of disutilities related to cancer screening programs should take into consideration the evaluation instruments used and other methodological differences among the studies. With regard to the DCE, because of the methodological differences, we could not compare the retrieved disutility values with other evaluation approaches. Despite a few disadvantages (potential underlying mismatch with random utility theory, irrational respond issue and difficulty of incorporating QALYs values), DCE has multiple benefits such as a trade-off between options, less cognitive burden, easier administration, and less measurement error.[16, 71] Stolk *et al* (2010) proposed a hybrid of TTO and DCE, [72] which could maximize the advantages of both methods enabling the precision utility elicitation. We believe this might be a promising strategy to follow in future studies assessing disutility of cancer screening programs.

### Impact of the research findings

Our findings suggest that disutilities related to cancer screening are mainly related to diagnostic work up though these results are uncertain because of either not reported or wide confidence intervals. The disutilities related to treatment phase are not explored. While on population scale the screening phase is the most important for disutilities assessment, since it affects each screened individual, all high quality studies report zero disutility on this stage (four studies report zero and one includes zero into the range of values). If high quality studies are used as a reference point, economic evaluations reporting estimated disutilities relevant to screening stage overestimate their values. This will lead to overestimation in cost-effectiveness ratio of cancer screening programs. Besides, when applying probabilistic sensitivity analyses where utilities and disutilities are assumed to be independent, this assumption will increase the uncertainty regarding incremental cost-effectiveness estimates.

### Limitations

This review is subject to several limitations. First of all, the applied quality criteria need further validation on the other studies. In addition, given very limited data retrieved per each typology and heterogeneity of the results, meta-synthesis was not feasible. [73] Considering incomparability between DCE and other direct or indirect methods, we could not incorporate these values into qualitative synthesis. Lastly, our inclusion criteria were limited to English-language articles only, which may not identify all the relevant studies.

### Research gap

To conclude, further research is needed in the area of disutility assessment. From all the typologies, the priority should be targeted at the potential moderate level of harms (false-positive diagnosis and overtreatment). More studies are necessary to assess disutility related to colorectal, lung and prostate cancer screening.

Furthermore, our review identified that around 60% of authors estimated the time frame for certain health state in their utility studies; therefore, we call for the urgent needs to standardize the time frame reporting. Lastly, given the advantage of allowing trade-off between options of DCE method, we think it is valuable to introduce more DCE studies in cancer screening programs. Such approach will help to improve the evidence for cost utility analysis and to facilitate further the sound decision making process for cancer screening programs.

## Conclusion

Cancer screening programs lead to low disutities related to screening phase, and low to moderate disutilities related to diagnostic work up and treatment phases. These disutility values varied by the measurement instrument applied and study quality.

## Supporting information

S1 Checklist PRISMA 2009 checklist. (PDF)

S1 File Appendix 1. (PDF)

S2 File Appendix 2. (PDF)

S1 Table Data extraction form of publications with measured disutility value. (PDF)

S2 Table Data extraction form of publications with estimated disutility values. (PDF)

S3 Table Summary table of quality appraisal. (PDF)

## References

[1] Forman D, Bray F, Brewster DH, Mbalawa CG, Kohler B, Pineros M, et al Cancer incidence in five continents Vol X IARC Scientific Publications No. 164. International Agency for Research on Cancer; 2014.

[2] Freddie B, Jacques F, Isabelle S, Rebecca LS, Lindsey AT, Ahmedin J. Global Cancer Statistics 2018: GLOBOCAN Estimates of Incidence and Mortality Worldwide for 36 Cancersin 185 Countries. CA CANCER J CLIN. 2018;0:1–31.

[3] Pace LE, Keating NL. A systematic assessment of benefits and risks to guide breast cancer screening decisions. JAMA. 2014;311(13):1327–1335. 10.1001/jama.2014.1398 24691608

[4] Moyer VA. Screening for Cervical Cancer: U.S. Preventive Services Task Force Recommendation Statement. Ann Intern Med. 2012:156:880–891. 10.7326/0003-4819-156-12-201206190-00424 22711081

[5] Lauby-Secretan B, Vilahur N, Bianchini F, Guha N, Straif K. The IARC Perspective on Colorectal Cancer Screening. NEJM. 2018;378(18): 1734–40. 10.1056/NEJMsr1714643 29580179 PMC6709879

[6] Chudgar NP, Bucciarelli PR, Jeffries EM, Rizk NP, Park BJ, Adusumilli PS, et al Results of the National Lung Cancer Screening Trial: Where Are We Now? Thorac Surg Clin. 2015; 25(2): 145–153. 10.1016/j.thorsurg.2014.11.002 25901558 PMC4817217

[7] Woolf SH, Harris R. The Harms of Screening New Attention to an Old Concern. JAMA. 2012;307 (6):565–567. 10.1001/jama.2012.100 22318274

[8] Alibhai SMH. Cancer screening: The importance of outcome measures. Critical Reviews in oncology/Hematology. 2006; 57: 215–224. 10.1016/j.critrevonc.2005.08.002 16371251

[9] Harris RP, Sheridan SL, Lewis CL, Barclay C, Vu MB, Kistler CE, et al The harms of screening: a proposed taxonomy and application to lung cancer screening. JAMA Intern Med. 2014;174:281–5. 10.1001/jamainternmed.2013.12745 24322781

[10] Sharp L, Cotton S, Carsin AE, Gray N, Thornton A, Cruickshank M, et al; on behalf of the TOMBOLA Group. Factors associated with psychological distress following colposcopy among women with low-grade abnormal cervical cytology: a prospective study within the Trial Of Management of Borderline and Other Low-grade Abnormal smears (TOMBOLA). Psychooncology. 2013;22(2):368–80. 10.1002/pon.2097 22162138

[11] Esserman LJ, Thompson IM Jr, Reid B. Overdiagnosis and Overtreatment in Cancer An Opportunity for Improvement. JAMA. 2013; 310 (8) 797–798. 10.1001/jama.2013.108415 23896967

[12] Harris RP, Wilt TJ, Qaseem A. A Value Framework for Cancer Screening: Advice for High-.Value Care From the American College of physicians. Ann Intern Med. 2015;162:712–717. 10.7326/M14-2327 25984846

[13] Wilt TJ, Harris RP, Qaseem A. Screening for Cancer: Advice for High-Value Care From the American College of Physicians. Ann Intern Med. 2015;162:718–725. 10.7326/M14-2326 25984847

[14] Woolf SH, Harris R. The Harms of Screening New Attention to an Old Concern. JAMA. 2012;307 (6):565–567. 10.1001/jama.2012.100 22318274

[15] Peasgood T, Ward SE, Brazier J. Health state utility values in breast cancer. Expert Rev Pharmacoeconomics outcomes Res. 2010; 10(5): 553–566. 10.1586/erp.10.65 20950071

[16] Ali S, Ronaldson S. Ordinal preference elicitation methods in health economics and health services research: using discrete choice experiments and ranking methods. British Medical Bulletin. 2012; 103: 21–44. 10.1093/bmb/lds020 22859714

[17] Drummond MF, Sculpher MJ, Claxton K, Stoddart GL, Torrance GW. Methods for the economic evaluation of health care programs 4th edition Oxford University Press; 2015 pp.133.

[18] Torrance GW, Feeny D. Utility and quality adjusted life years. Int J of Technology Assessment in Health Care. 1989;5: 559–575. 10.1017/s0266462300008461 2634630

[19] Djalalov S, Rabeneck L, Tomlinson G, Bremner KE, Hilsden R, Hoch JS. A Review and Meta-analysis of Colorectal Cancer Utilities. Med Decis Making. 2014;34:809–818. 10.1177/0272989X14536779 24903121

[20] Schiller-Fru¨hwirth IC, Jahn B, Arvandi M, Siebert U. Cost-Effectiveness Models in Breast Cancer Screening in the General Population: A Systematic Review. Appl Health Econ Health Policy. 2017; 15:333–351. 10.1007/s40258-017-0312-3 28185134

[21] Earle CC, Chapman RH, Baker CS, Bell CM, Stone PW, Sandberg EA, et al Systematic Overview of Cost-Utility Assessments in Oncology. J Clin Oncol. 2000; 18:3302–3317. 10.1200/JCO.2000.18.18.3302 10986064

[22] Ara R, Brazier J, Peasgood T, Paisley S. The identification, review and synthesis of health state utility values from the literature. PharmacoEconomics. 2017; 35 (Suppl 1):S43–S55. 10.1007/s40273-017-0547-8 29052156

[23] Papaioannou D, Brazier J, Paisley S. Systematic Searching and Selection of Health State Utility Values from the Literature. Value in Health. 2013;16:686–695. 10.1016/j.jval.2013.02.017 23796303

[24] De Kok IMCM Korfage IJ, van den Hout WB, Helmerhorst TJM, Habbema JDF, Essink-Bot ML. Quality of life assumptions determine which cervical cancer screening strategies are cost-effective. Int J Cancer. 2018;142, 2383–2393. 10.1002/ijc.31265 29349795

[25] Insinga RP, Glass AG, Myer ERs, Rush BB. Abnormal Outcomes Following Cervical Cancer Screening: Event Duration and Health Utility Loss. Med Decis Making. 2007;27:414–422. 10.1177/0272989X07302128 17585005

[26] De Haes JCJM, de Koning HJ, van Oortmarssen GJ, van Agt HME, de Bruyn AE, vander Masa PJ. A impact of breast cancer screening program on quality adjusted life years. Int J Cancer. 1991;49:538–44. 10.1002/ijc.2910490411 1917155

[27] De Koning HJ, van Ineveld BM, van Oortmarssen GJ, de Haes JCJM, Collette HJA, Hendriks JHCL, et al Breast cancer screening and cost effectiveness: policy alternatives, quality of life considerations and the possible impact of uncertain factors. Int J Cancer. 1991;49:531–37. 10.1002/ijc.2910490410 1917154

[28] Bonomi AE, Boudreau DM, Fishman PA, Ludman E, Mohelnitzky A, Cannon EA, et al Quality of life valuations of mammography screening. Qual Life Res. 2008; 17:801–814. 10.1007/s11136-008-9353-2 18491217

[29] Essink-Bot ML, de Koning HJ, Nijs HGT, Kirkels WJ, van der PJ, Schroder MF. Short-Term Effects of Population-Based Screening for Prostate Cancer on Health-Related Quality of Life. J Natl Cancer Inst. 1998;90(12) 925–31. 10.1093/jnci/90.12.925 9637143

[30] Cormier L, Guillemin F, Valerl A, Fournier G, Cussenot O, Mangin P, et al Impact of prostate cancer screening on health related quality of life in high risk families. Urology. 2002;59:901–906. 10.1016/s0090-4295(02)01552-2 12031378

[31] Vasarainen H, Malmi H, Määttänen L, Ruutu M, Tammela T, Taari K, et al Effects of prostate cancer screening on health related quality of life: Results of the Finnish arm of the European randomized screening trial (ERSPC). Acta Oncologica. 2013;52: 1615–21. 10.3109/0284186X.2013.802837 23786174

[32] Tosteson ANA, Fryback DG, Hammond CS, Hanna LG, Grove MR, Brown M, et al Consequences of False-Positive Screening Mammograms. JAMA Intern Med. 2014;174(6):954–961. 10.1001/jamainternmed.2014.981 24756610 PMC4071565

[33] Gerard K, Johnston K, Brown J. The role of a pre-scored multi-attribute health classification measure in validating condition specific health state descriptions. Health Econ.1999;8: 685–99. 10590470 10.1002/(sici)1099-1050(199912)8:8<685::aid-hec472>3.0.co;2-8

[34] Johnston K, Brown J, Gerard K, O'hanlon M, Morton A. Valuing temporary ad chronic health states associated with breast screening. Soc Sci Med. 1998;47(2): 213–222. 9720640 10.1016/s0277-9536(98)00065-3

[35] Gyrd-hansen D, Sogaard J. Analysing public preference for cancer screening programs. Health Econ. 2001; 10: 617–634. 11747045 10.1002/hec.622

[36] Rijnsburger AJ, Essink-Bot ML, van Dooren S, Borsboom GJJM, Seynaeve C, Bartels CCM, et al Impact of screening for breast cancer in high-risk women on health-related quality of life. British J of cancer. 2004;91:69–76. 10.1038/sj.bjc.6601912 PMC2364757 15199386

[37] Howard K, Salkeld G, Mccaffery K, Irwig L. HPV triage testing for repeat pap smear for the management of atypical squamous cells(ASCUS) on pap smear: is there evidence of process utility? Health Econ. 2008;17:593–605. 10.1002/hec.1278 17764095

[38] Birch S, Melnikow J, Kuppermann M. Conservative versus aggressive follow up of mildly abnormal Pap smears: Testing for process utility. Health Econ. 2003;12:879–84. 10.1002/hec.783 14508872

[39] Melnkow J, Kuppermannm M, Birch S, Chan BS, -Nuovo J. Management of the low-grade abnormal Pap smear: What are women’s preferences? The Journal of Family practice. 2002;51(10): 849–855. 12401153

[40] Whynes DK, Woolley C, Philip Z. Management of low-grade cervical abnormalities detected at screening: which method do women prefer? Cytopathology.2008;19:355–362. 10.1111/j.1365-2303.2008.00565.x 18522634

[41] TOMBOLA group. Options for managing low grade cervical abnormalities detected at screening: cost effectiveness study. BMJ. 2009;339:1–7. 10.1136/bmj.b2549 PMC2718086 19638648

[42] Howard K, Salkeld G. Does Attribute Framing in Discrete Choice Experiments Influence Willingness to Pay? Results from a discrete choice experiment in screening for colorectal cancer. Value in health. 2009;12(2):354–63. 10.1111/j.1524-4733.2008.00417.x 18657102

[43] Marshall DA, Johnson FR, Kulin NA, Özdemir S, Walsh JM, Marshall JK. How do physician assessments of patient preferences for colorectal cancer screening tests differ from actual preferences? A comparison in Canada and the United States using a stated choice survey. Health econ. 2009;18(12): 1–26. 10.1002/hec.1437 PMC3964796 19191268

[44] Drolet M, Brisson M, Maunsell E, Franco EL, Coutlée F, Ferenczy A, et al The psychosocial impact of an abnormal cervical smear result. Psychooncology. 2012;21: 1071–81. 10.1002/pon.2003 21695747

[45] Simonella L, Howard K, Canfell K. A survey of population-based utility scores for cervical cancer prevention. BMC research notes. 2014;7:899–910. 10.1186/1756-0500-7-899 25495005 PMC4307910

[46] Stratton KR, Durch JS, Lawrence RS. Vaccines for the 21st Century: A Tool for Decision making. National Academy of Sciences; 2000 pp.215. 25121214

[47] Maissi E, Marteau TM, Hankins M, Moss S, Legood R, Gray A. The psychological impact of human papillomavirus testing in women with borderline or mildly dyskaryotic cervical smear test results: 6-month follow-up. British Journal of Cancer. 2005; 92: 990–994. 10.1038/sj.bjc.6602411 15785734 PMC2361952

[48] Mo X, Tobe RG, Wang L, Liu X, Wu B, Luo H, et al Cost-effectiveness analysis of different types of human papillomavirus vaccination combined with a cervical cancer screening program in mainland China. BMC Infection Diseases. 2017;17:502–51. 10.1186/s12879-017-2592-5 PMC5516327 28720082

[49] Ock M, Park JY, Son WS, Lee HJ, Kim SH, Jo MW. Estimation of utility weights for human papilloma virus-related health states according to disease severity. Health and quality of life outcomes. 2016;14:163–172. 10.1186/s12955-016-0566-8 27894347 PMC5126850

[50] Cantor SB, Volk RJ, Krahn MD, Cass AR, Gilani J, Weller SC, et al Concordance of couples’ prostate cancer screening recommendations form a decision analysis. Patient. 2008;1 (1):11–19. 10.2165/01312067-200801010-00004 22272754

[51] van Hees F, Habbema JD, Meester RG, Lansdorp- Vogelaar I, van Ballegooijen M, Zauber AG. Should Colorectal Cancer Screening Be Considered in Elderly Without Prior Screening? A Cost-Effectiveness Analysis. Ann Intern Med. 2014;160(11):750–759. 10.7326/M13-2263 24887616 PMC4109030

[52] Goede SL, Rabeneck L, van Ballegooijen M, Zauber AG, Paszat LF, Hoch JS. Harms, benefits and costs of fecal immunochemical testing versus guaiac fecal occult blood testing for colorectal cancer screening. PLOS one. 2017;12(3):1–15. 10.1371/journal.pone.0172864 PMC5351837 28296927

[53] Naber SK, Kuntz KM, Henrikson NB, Williams MS, Calonge N, Goddard KB, et al Cost effectiveness of age-Specific screening intervals for people with family histories of colorectal cancer. Gastroenterology. 2018;154:105–116. 10.1053/j.gastro.2017.09.021 28964749 PMC6104831

[54] De Bekker-Grob EW, de Kok IMCM, Bulten J, van Rosmalen J, Vedder JEM, Arbyn M, et al Liquid-based cervical cytology using ThinPrep technology: weighing the pros and cons in a cost-effectiveness analysis. Cancer Causes Control. 2012: 23:1323–1331. 10.1007/s10552-011-9850-4 22706692

[55] van Rosmalen J, de Kok IMCM, van Ballegooijen M. Cost-effectiveness of cervical cancer screening: cytology versus human papillomavirus DNA testing. BJOG. 2012;119:699–709. 10.1111/j.1471-0528.2011.03228.x 22251259 PMC3489039

[56] Pataky R, Phillips N, Peacock S, Coldman AJ. Cost-effectiveness of population-based mammography screening strategies by age range and frequency. Journal of cancer policy. 2014;2:97–102.

[57] Stout NK, Rosenberg MA, Trentham-Dietz A, Smith MA, Robinson SM, Fryback DG. Retrospective cost-effectiveness analysis of screening mammography. Journal of national cancer institute. 2006;98(11): 774–782. 10.1093/jnci/djj210 16757702

[58] Kitchener HC, Blanks R, Cubie H, Desai M, Legood GDR, Gray A, et al MAVARIC–a comparison of automation-assisted and manual cervical screening: a randomized controlled trial. Health Technology Assessment. 2011; 15(3):1–176. 10.3310/hta15030 21266159

[59] Manser R, Dalton A, Carter R, Byrnes G, Elwood M, Campbell DA. Cost-effectiveness analysis of screening for lung cancer with low dose spiral CT (computed tomography) in the Australian setting. Lung Cancer. 2005; 48, 171–185. 10.1016/j.lungcan.2004.11.001 15829317

[60] Raab SS, Hornberger J. The effect of a patient's risk-taking attitude on the cost effectiveness of testing strategies in the evaluation of pulmonary lesions. Chest. 1997;111:1583–1590. 10.1378/chest.111.6.1583 9187178

[61] Berkhof J, Coupe VM, Bogaards JA, van Kemenade FJ, Helmerhorst TJ, Snijders PJ. The health and economic effects of HPV DNA screening in the Netherlands. Int J Cancer. 2010;127, 2147–2158. 10.1002/ijc.25211 20112339

[62] Thomson MD, Siminoff LA. Perspectives on Mammography after Receipt of Secondary Screening Owing to a False Positive. Women's Health Issues. 2015;25 (2); 128–133. 10.1016/j.whi.2014.11.003 25648490 PMC4355242

[63] Havrilesky L, Gierisch JM, Moorman P, McCrory D, Ghate S, Williams J, et al Systematic Review of Cancer Screening Literature for Updating American Cancer Society Breast Cancer Screening Guidelines Duke Evidence Synthesis Group for American Cancer Society, 2014

[64] Myers ER, Moorman P, Gierisch JM, Havrilesky LJ, Grimm LJ, Ghate S, et al Benefits and Harms of Breast Cancer Screening: A Systematic Review. JAMA. 2015;314: 1615–34. 10.1001/jama.2015.13183 26501537

[65] Armstrong K, Moye E, Williams S, Berlin JA, Reynolds EE. Screening mammography in women 40 to 49 years of age: a systematic review for the American College of Physicians. Ann Intern Med. 2007;146: 516–26. 10.7326/0003-4819-146-7-200704030-00008 17404354

[66] Carter GC, King DT, Hess LM, Mitchell SA, Taipale KL, Kiiskinen U, et al Health stateutility values associated with advanced gastric, oesophageal, or gastrooesophageal junction adenocarcinoma: a systematic review. Journal of Medical Economics. 2015;18(11): 954–966. 10.3111/13696998.2015.1066380 26212479

[67] Hanmer J, Lawrence WF, Anderson JP, Kaplan RM, Fryback DG. Report of Nationally Representative Values for the Noninstitutionalized US Adult Population for 7 Health-Related Quality-of-Life Scores. Med Decis Making. 2006;26:391–400. 10.1177/0272989X06290497 16855127

[68] Kim SH, Jo MW, Ock M, Lee HJ, Lee JW. Estimation of health state utilities in breast cancer. Patient Preference and Adherence. 2017:11 531–536. 10.2147/PPA.S129856 28352159 PMC5359127

[69] Bremner KE, Chong CAKY, Tomlinson G, Alibhai SMH, Krahn MD. A Review and Meta-Analysis of Prostate Cancer Utilities. Med Decis Making. 2007;27:288–298. 10.1177/0272989X07300604 17502448

[70] Green C, Brazier J, Deverill M. Valuing Health-Related Quality of Life A Review of Health State Valuation Techniques. Pharmacoeconomics. 2000; 17 (2): 151–165. 10.2165/00019053-200017020-00004 10947338

[71] Bansback N, Brazier J, Tsuchiya A, Anis A. Using a discrete choice experiment to estimate societal health state utility values. Discussion Paper. HEDS Discussion Paper 10/03. (Unpublished) 2010. [cited 2018 May 19] Available from: http://eprints.whiterose.ac.uk/10900/.

[72] Stolk EA, Oppe M, Scalone L, Krabbe PFM. Discrete Choice Modeling for the Quantification of Health States: The Case of the EQ-5D. Value in health. 2010; 13(8): 1005–1013. 10.1111/j.1524-4733.2010.00783.x 20825618

[73] Torrance GW, Furlong W, Feeny D. Health utility estimation. Expert Review of Pharmacoeconomics & Outcomes Research. 2002; 2 (2): 99–108. 19807322 10.1586/14737167.2.2.99

