# Supplementary material for: Disutility associated with cancer screening programs: A systematic review
Source: PLoS One. 2019 Jul 24;14(7):e0220148. doi: 10.1371/journal.pone.0220148 (PMC6655768; doi:10.1371/journal.pone.0220148)
Supplement: S1 File — (PDF) [file pone.0220148.s002.pdf]

Appendix 1:

embase.com

(((neoplasm/de OR breast tumor/exp OR 'large intestine cancer'/exp OR 'lung tumor'/exp OR 'prostate cancer'/exp OR 'uterine cervix cancer'/exp OR 'intestine endoscopy'/exp OR 'breast examination'/exp OR 'Papanicolaou test'/exp OR 'Wart virus'/de OR 'prostate specific antigen'/de OR 'occult blood'/de OR 'occult blood test'/de) AND ('mass screening'/de OR 'cancer screening'/de OR screening/de OR 'screening test'/de OR 'early cancer diagnosis'/de)) OR (((colonoscop* OR sigmoidoscop* OR mammograph* OR colposcop* OR Papanicolaou OR pap-smear* OR hpv OR 'Wart virus*' OR 'prostate specific antigen*' OR psa OR ldct OR low-dose-ct OR low-dose-comput* OR 'occult blood' OR fobt OR fecal OR faecal OR feces OR faeces) NEAR/10 (screen*)) OR ((cancer* OR neoplas* OR malign* OR carcinom*) NEAR/3 (breast OR lung OR colorectal* OR cervical* OR 'uterine cervix' OR prostat*) NEAR/10 (screen*))):ab,ti,kw) AND ('quality adjusted life year'/de OR 'disability-adjusted life year'/de OR 'cost utility analysis'/de OR ('life expectancy'/de AND ('quality of life'/de OR 'disability'/de OR 'health status'/de)) OR (((qualit* OR disabilit*) NEAR/3 adjust* NEAR/3 (life-year* OR life-expect* OR lifeyear*)) OR ((gain* OR 'health stat*' OR disabilit* OR qualit* OR qol) NEAR/6 life NEXT/1 (year* OR expect*)) OR QALY* OR QALE OR QALEs OR DALY* OR utilit* OR disutilit*):ab,ti,kw) NOT ([Conference Abstract]/lim OR [Letter]/lim OR [Note]/lim OR [Editorial]/lim) AND [english]/lim

Medline Ovid

(((neoplasms/ OR exp Breast Neoplasms/ OR exp Colorectal Neoplasms/ OR exp Lung Neoplasms/ OR exp Prostatic Neoplasms/ OR exp Uterine Cervical Neoplasms/ OR Colonoscopy/ OR Mammography/ OR Papanicolaou Test/ OR Papillomaviridae/ OR Prostate-Specific Antigen/ OR Occult Blood/) AND (Mass Screening/ OR Early Detection of Cancer/)) OR (((colonoscop* OR sigmoidoscop* OR mammograph* OR colposcop* OR Papanicolaou OR pap-smear* OR hpv OR Wart virus* OR prostate specific antigen* OR psa OR ldct OR low-dose-ct OR low-dose-comput* OR occult blood OR fobt OR fecal OR faecal OR feces OR faeces) ADJ10 (screen*)) OR ((cancer* OR neoplas* OR malign* OR carcinom*) ADJ3 (breast OR lung OR colorectal* OR cervical* OR uterine cervix OR prostat*) ADJ10 (screen*))).ab,ti,kw) AND (Quality-Adjusted Life Years/ OR (Life Expectancy/ AND (Quality of Life/ OR Health Status/)) OR (((qualit* OR disabilit*) ADJ3 adjust* ADJ3 (life-year* OR life-expect* OR lifeyear*)) OR ((gain* OR health stat* OR disabilit* OR qualit* OR qol) ADJ6 life ADJ (year* OR expect*)) OR QALY* OR QALE OR QALEs OR DALY* OR utilit* OR disutilit*).ab,ti,kw) NOT (letter* OR news OR comment* OR editorial* OR congres* OR abstract* OR book* OR chapter* OR dissertation abstract*).pt. AND english.la.

Cochrane CENTRAL

((((colonoscop* OR sigmoidoscop* OR mammograph* OR colposcop* OR Papanicolaou OR pap-smear* OR hpv OR 'Wart virus*' OR 'prostate specific antigen*' OR psa OR ldct OR low-dose-ct OR low-dose-comput* OR 'occult blood' OR fobt OR fecal OR faecal OR feces OR faeces) NEAR/10 (screen*)) OR ((cancer* OR neoplas* OR malign* OR carcinom*) NEAR/3 (breast OR lung OR colorectal* OR cervical* OR 'uterine cervix' OR prostat*) NEAR/10 (screen*))):ab,ti,kw) AND ((((qualit* OR disabilit*) NEAR/3 adjust* NEAR/3 (life-year* OR life-expect* OR lifeyear*)) OR ((gain* OR 'health stat*' OR disabilit* OR qualit* OR qol) NEAR/6

life NEXT/1 (year* OR expect*)) OR QALY* OR QALE OR QALEs OR DALY* OR utilit* OR disutilit*):ab,ti,kw)

Web of science

TS=(((((colonoscop* OR sigmoidoscop* OR mammograph* OR colposcop* OR Papanicolaou OR pap-smear* OR hpv OR "Wart virus*" OR "prostate specific antigen*" OR psa OR ldct OR low-dose-ct OR low-dose-comput* OR "occult blood" OR fobt OR fecal OR faecal OR feces OR faeces) NEAR/10 (screen*)) OR ((cancer* OR neoplas* OR malign* OR carcinom*) NEAR/2 (breast OR lung OR colorectal* OR cervical* OR "uterine cervix" OR prostat*) NEAR/10 (screen*)))) AND ((((qualit* OR disabilit*) NEAR/2 adjust* NEAR/2 (life-year* OR life-expect* OR lifeyear*)) OR ((gain* OR "health stat*" OR disabilit* OR qualit* OR qol) NEAR/5 life NEAR/1 (year* OR expect*)) OR QALY* OR QALE OR QALEs OR DALY* OR utilit* OR disutilit*))) AND DT=(article) AND LA=(english)

Google scholar

colonoscopy|sigmoidoscopy|mammography|colposcopy|Papanicolaou|"pap-smear"|"prostate specific antigen" |"low-dose-ct|computed"|"occult blood"|"breast|lung|colorectal|cervical |cervix|prostate cancer|neoplasms|carcinoma" screening QALY|DALY|utility|disutility
