# Supplementary material for: Disutility associated with cancer screening programs: A systematic review
Source: PLoS One. 2019 Jul 24;14(7):e0220148. doi: 10.1371/journal.pone.0220148 (PMC6655768; doi:10.1371/journal.pone.0220148)
Supplement: S2 File — (PDF) [file pone.0220148.s003.pdf]

Appendix 2:

- Centre for Reviews and Dissemination databases: NHS EED and HTA
  https://www.crd.york.ac.uk/CRDWeb/
- Tufts Cost-effectiveness Analysis Registry (former Harvard Cost-Effectiveness Analysis database)
  http://healtheconomics.tuftsmedicalcenter.org/cear4/Home.aspx
- Nice Decision Support Unit
  http://nicedsu.org.uk/
- CADTH
  https://www.cadth.ca
- ISOQOL
  http://www.isoqol.org/
- Eunethta
  https://www.eunethta.eu/
- ISPOR
  https://www.ispor.org/
