# Supplementary material for: Disutility associated with cancer screening programs: A systematic review
Source: PLoS One. 2019 Jul 24;14(7):e0220148. doi: 10.1371/journal.pone.0220148 (PMC6655768; doi:10.1371/journal.pone.0220148)
Supplement: S1 Table — (PDF) [file pone.0220148.s004.pdf]

Data extraction form of publications with measured disutility value

Author<br>Publication Year	Study Overview<br>(a, Study design; b) Aim;<br>c. Country)	Cancer Type	Intervention	Instrument used for utility measurement	Respondents<br>(N=sample size)	Disutility Typology	Disutility Value<br>Mean (95%CI if reported)	Time Frame<br>（A=assumption, G=guideline, M=measurement）	QALY[#]<br>or other findings	Quality Assessment Result
Birch S, 2002	-Cohort study<br>- Utility of conservative vs. aggressive follow up for mildly abnormal Pap smears subjects<br>-USA	Cervical cancer	Diagnostic:<br>-Pap smear<br>-colposcopy	SG	Women with mildly abnormal pap smears (N=148)	Diagnostic work up phase<br>Repeat pap smear (no pathology)	0.042	18 months (G)	Aggressive vs. conservative protocol: 0.083 QALY loss	Low
						Diagnostic work up phase<br>Early colposcopy(no pathology)	0.073	24 months (G)		
Bonomi AE, 2008	-Cohort study (randomly sampled from BCSP (no comparator)<br>- QOL valuation<br>-USA	Breast cancer	-Screening:-mammography<br><br>Diagnostic:<br>-MM<br>-US<br>-needle biopsy	VAS	Women aged 50-79years (N=131)	Screening phase	0.20	Up to 3 weeks (A)	0.012 QALY loss	Low
						Diagnostic work up phase<br>False positive	0.45<br><br>0.19	2 weeks (A)	0.017 QALY loss<br><br>0.007 QALY loss	
Cantor SB, 2008	-Cross-sectional<br>- Couples' preference for prostate cancer screening outcomes<br>-USA	Prostate cancer	PSA	TTO	Married couples from family practice center (N=336)	Treatment phase<br>overtreatment	0.09-0.27 (couples' value, includes impotence, urinary incontinence, injury outcomes)	Remaining years of life expectancy (M)	Husbands estimate the disutility value higher than wives<br>0.09-0.27 QALYs loss	Medium
Cormier L, 2002	-Cohort study<br>-Screening impact on the quality of life for high risk men<br>-France	Prostate cancer	-PSA	RAND SF-36	Men aged 40-70 with family history of prostate cancer (N=220)	Screening phase	0-0.03<br>(4 out of 8 dimensions shown disutility)	15.6 days (M)	0-0.0013 QALY loss	High
De Kok IMCM 2018	-Cohort study<br>-Cost-effectiveness of screening strategies<br>-the Netherlands	Cervical cancer	Screening:<br>-Pap smear<br>Diagnostic:<br>-Pap smear<br>-HPV<br>-Colposcopy	-SF-6D<br>-EQ-5D	Women invited to screening till diagnosis (N=3087)	Screening phase	Attendees vs reference group there is no disutility found (0.02 – 0.03 higher in screened)	Not available	Both SF-6D and EQ-5D utility value higher from screening participant vs reference group.	High
						Diagnostic work up phase<br>Repeat Pap smear	0.03 (SF-6D),no disutility (EQ-5D)	6-24 months (G)	For SF-6D mean 0.0375 QALY loss	
						Colposcopy referral	0.01-0.02(SF-6D), no disutility (EQ-5D)	6 months (G)	For SF-6D 0-0.01 QALY loss	

Author<br>Publication Year	Study Overview<br>(a, Study design; b) Aim; c. Country)	Cancer Type	Intervention	Instrument used for utility measurement	Respondents<br>(N=sample size)	Disutility Typology	Disutility Value<br>Mean (95%CI if reported)	Time Frame<br>（A=assumption, G=guideline, M=measurement）	QALY[#]<br>or other findings	Quality Assessment Result
Drolet M, 2012	-Controlled study<br>- Psychological impact of abnormal screening result<br>-Canada	Cervical cancer	-Cervical smear	-EQ-5D<br>-VAS<br>-SF-6D	Women (over 18 years old; N=492 with abnormal smear result; N=460 with normal result)	Diagnostic work up phase<br>All abnormal cases vs normal cases	Baseline: EQ-5D 0.0269; VAS 0.0277; SF-6D 0.026<br>12 weeks: EQ-5D 0.0163;VAS 0.0183;SF-6D 0.025	0-12 weeks (M; from 1st screening result ready onward)	No QALY loss at 12 week	High
Essink-Bot ML, 1998	-Longitudinal controlled study<br>- short term effect of screening and biopsy on health status<br>-the Netherlands	Prostate cancer	Screening:<br>-PSA<br>Diagnostic:<br>-DRE<br>-TRUS<br>-biopsy	-SF-36<br>-EQ-5D	Men aged 55-74 years old (N=626 screening attenders N=500 nonparticipants)	Screening phase<br><br>Diagnostic work up phase	No disutility found among attendees vs nonparticipants (the utility value is higher in screened group for 8 dimensions of SF-36 ; 0.02 higher in EQ-5D)	T1(3 weeks before initial visit)till T5 (1 week after confirmation of no cancer in case of FP result) (M)	No QALY loss	High
Gerard K, 1999	-Cohort study<br>-Compare the values from different method<br>-UK	Breast cancer	-Not specified	EuroQol<br>TTO	Women from breast screening center (N=440)	Diagnostic work up phase<br>False positive	Short term: 0.15 (EuroQol, vs TN)<br>0.26 (TTO, vs TN);<br>0.65 (vs full health)	12 month (A)	0.15 (EuroQol, vs TN)<br>0.26 (TTO, vs TN);<br>0.65 (vs full health)	Medium
Gyrd-Hansen D, 2001	-Modeling study<br>-Utility function for cancer screening (both utility and disutility perspectives)<br>-Denmark	Breast cancer	Mammogram	Discrete ranking modeling	Women aged 50 years old (N= 255)	Diagnostic work up phase<br>False positive	Disutility demonstrated (beta coefficient= − 0.000297 ;p= 0.0162)	Not applicable	A significant negative coefficient for false positive (squared FP) is found which indicates the increasing marginal disutility of a false positive diagnosis in breast cancer screening.	High
		Colorectal cancer	Screening:<br>-stool test<br>Diagnostic:<br>-colonoscopy	Discrete ranking modeling	Men and women aged 50 years old (N=509)	Diagnostic work up phase<br>False positive	No disutility demonstrated	Not applicable	False positive diagnosis has no impact in utility value for colorectal cancer screening	
Haes JCJM, 1991	-Modeling study<br>-QALY of breast cancer screening program<br>-the Netherlands	Breast cancer	-Mammogram	VAS	Professionals (Employees from Dept. of Public Health N=15; Experts in breast cancer	Screening phase	0.006	1 week (A)	0.00012 QALY loss	Low
						Diagnostic work up phase	0.105	5 week (A)	0.010 QALY loss	

Author Publication Year	Study Overview (a, Study design; b) Aim; c. Country)	Cancer Type	Intervention	Instrument used for utility measurement	Respondents (N=sample size)	Disutility Typology	Disutility Value Mean (95%CI if reported)	Time Frame （A=assumption, G=guideline, M=measurement）	QALY# or other findings	Quality Assessment Result
					N=12)					
Howard K, 2008	--Cohort study<br>-Utility of HPV triage test vs repeat Pap smears for ASCUS subjects<br>-Australia	Cervical cancer	-Pap smears<br>-HPV	SG	Women aged 18- 70 years (N=73 at baseline and N=67 at 7 weeks)	Diagnostic work up phase<br>Repeat Pap smear	0.0028(0.0020-0.0036)	18 months (G)	For spontaneous resolution/HPV negative group the aggressive protocol lead to 0.0008 QALY loss	Medium
						Diagnostic work up phase<br>Immediate HPV	0.0033(0.0022-0.0043)	18 months (G)		
Howard K, 2009	-Modeling study<br>-Preferences in colorectal cancer screening<br>-Australia	Colorectal cancer	Screening:<br>-FOBT<br>Diagnostic:<br>-Colonoscopy	DCE	General population (N=1920)	Diagnostic work up phase	Disutility demonstrated for unnecessary colonoscopy (beta-coefficient=-0.01-0.02; P <0.00001)and non-accuracy of tests (beta-coefficient=-0.06-0.21; P <0.00001)	Not applicable	Accuracy of tests more likely to influence the choices	Medium
Insinga RP, 2007	--Retrospective cohort study<br>- QALY loss for abnormal outcomes from screening<br>-USA	Cervical cancer	Screening:<br>-Pap smear<br>Diagnostic:<br>-biopsy	Estimation	Women with abnormal smear result (N=923)	Diagnostic work up phase<br>False positive	0.047 (calculated from QALY loss)	10.3 months(M)	0.04 QALY loss	Medium
						CIN1, CIN2 CIN3	0.067 (calculated from QALY loss)	17.7-21.6 Months (M)	0.11 QALY loss	
Myers ER, 2004	- Health state utility weights<br>-USA	Cervical cancer	Screening:<br>Pap smear cytology<br>Diagnostic:<br>biopsy	TTO	Female volunteer (N=150)	Screening phase	0.02	1-2 weeks (G)	0.0006 QALY loss	
						Diagnostic work up phase<br>ASC	0.06	Not available		
						LSIL/HSIL	0.09	Not available		
						CIN1	0.09	Not available		
						CIN 2/3	0.13	Not available		
Johnston K, 1998	-Cohort study<br>-Quality of life value for breast cancer screening<br>-UK	Breast cancer	-Not specified	TTO<br>VAS	Women aged 40-44 & 50-64 (N=440)	Diagnostic work up phase<br>False positive	0.25(0.22～0.28) (TTO, vs TN)<br>0.23(0.20～0.25) (VAS, vs TN)	12 month (A)	0.25 QALY loss (TTO)<br>0.23 QALY loss (VAS)	Medium
Koning HJ, 1991	-Modeling study<br>-Cost- effectiveness of five screening options<br>-the Netherlands	Breast cancer	Screening:<br>-Mammography<br>Diagnostic:	VAS	Clinician or public health expert (N=27)	Screening phase	0.01	1 week (A)	0.0002 QALY loss	Very Low
						Diagnostic work up phase	0.11	5 weeks (A)	0.011 QALY loss	

Author<br>Publication Year	Study Overview<br>(a, Study design; b) Aim; c. Country)	Cancer Type	Intervention	Instrument used for utility measurement	Respondents<br>(N=sample size)	Disutility Typology	Disutility Value<br>Mean (95%CI if reported)	Time Frame<br>（A=assumption, G=guideline, M=measurement）	QALY[#]<br>or other findings	Quality Assessment Result
			-Biopsy			False positive				
Maissi E, 2005	-Controlled cohort study (compare normal group vs BMD[14] group)<br>-Psychological impact of BMD result<br>-UK	Cervical cancer	-Pap<br>-HPV	EQ-5D	Women, N= 1376 (1 month) and N=1011 (6 months)	<u>Diagnostic work up phase</u><br>at 1 month	0.02-0.04	1 month (M)	Mean 0.0025 QALY loss	High
						at 6 months	BMD vs normal group there is no disutility found	6 month (M)	No QALY loss	
Marshall DA, 2009	-Modeling study<br>-Patient preferences for colorectal cancer screening<br>-USA & Canada	Colorectal cancer	-Stool test<br>-Colonoscopy/Bioscope<br>-CT<br>-Barium enema	DCE	General population aged 45-70 (Canada N=501; USA N=1087)<br>Physicians<br>(N=100, both US & Canada)	<u>Screening / Diagnostic work up phase</u>	Disutility demonstrated mainly from process （scope: beta coefficient=-0.11-0.14 ;P＜0.001 ）, mild pain （beta-coefficient=-0.26-0.28;p＜0.001）, complications(（1/100: beta coefficient =-0.20-0.24;p＜ 0.001） ， test sensitivity (（50%: beta-coefficient= -0.25,p＜0.001）, test specificity （40%: beta-coefficient=-0.32 -0.36;p＜0.001）	Not applicable	Top three contributor to disutility are: low test sensitivity(40%) , mild pain, and low test specificity (50%)for both Canada and US population	High
Melnikow J, 2002	-Cohort study<br>- reference for management of pap smear among women with low-grade abnormal result<br>-USA	Cervical cancer	-Pap smear<br>-colposcopy	SG	Women aged over 18 years (N=148)	<u>Diagnostic work up phase</u><br>Observation	0.04	18-36 months (A)	For spontaneous resolution group , aggressive protocol lead to 0.0657 QALY loss vs observation protocol	Low
						early colposcopy	0.07	18-36 months (A)		
Mo X, 2017 [##]	-Modeling study<br>- Cost effectiveness of HPV vaccination combined with screening program<br>-China	Cervical cancer	-LBC<br>-HPV DNA<br>-Pap smear cytology<br>-VIA	Scaling	Population based (number is not specified)	<u>Diagnostic work up phase</u><br>CIN1	0.01	1 year (A)	0.01 QALY loss	Very Low
						CIN2	0.12	1 year (A)	0.12 QALY loss	
						CIN3	0.19	1 year(A)	0.19 QALY loss	

Author Publication Year	Study Overview (a, Study design; b) Aim; c. Country)	Cancer Type	Intervention	Instrument used for utility measurement	Respondents (N=sample size)	Disutility Typology	Disutility Value Mean (95%CI if reported)	Time Frame （A=assumption, G=guideline, M=measurement）	QALY# or other findings	Quality Assessment Result
Ock M, 2016	-Cohort study<br>-Utility for HPV related health states<br>-Korea	Cervical cancer	HPV	SG<br>VAS	Women (≥19 years, N=900)	Diagnostic work up phase<br>HPV positive<br>condyloma<br>CIN I<br>CIN II-III	 <br>0.27 (VAS) 0.17(SG)<br>0.34 (VAS) 0.22 (SG)<br>0.39 (VAS) 0.23(SG)<br>0.40 (VAS) 0.23(SG)	Not available		Medium
Rijnsburger AJ, 2004	- Prospective observational study<br>- HRQoL impact on high risk women<br>-the Netherlands	Breast cancer	-Mammography +<br>MRI<br>+CBE +BSE	SF-36<br>EQ-5D	Women with high risk<br>(N=334)	Diagnostic work up phase	No disutility	1 or 4 weeks (M)	Compare with the reference group no disutility in both SF-36 & EQ-5D valuation	High
Simonella L, 2014	-Survey<br>-Utility score for HPV testing, cytology and HPV vaccination<br>-Australia	Cervical cancer	-HPV test<br>-Cytology	SG	General population aged 20-69 years<br>(N=43)	Diagnostic work up phase<br>Low grade disease	0.0265-0.0276	12 month (A)	0.0265-0.0276 QALY loss	Low
						HPV positive	0.0036-0.0267	12 month (A)	0.0036-0.0267 QALY loss	
						CIN1	0.0276	12 month (A)	0.0276 QALY loss	
						CIN2/3	0.0296	12 month (A)	0.0296 QALY loss	
Stratton KR, 2000	-A systematic quantitative research by IOM<br>- Generate the analytic model to evaluate vaccine program for decision making purpose<br>-USA	Cervical cancer	-Pap smears	HUI Mark II	IOM Committee	Diagnostic work up phase<br>Cervical dysplasia	0.03	18 months (G)	0.045 QALY loss	Low
TOMBOLA Group, 2009	-Randomized controlled trial<br>-Cost effectiveness of alternative methods of managing low grade cervical abnormalities<br>- UK	Cervical cancer	-Pap smear<br>-colposcopy	EQ-5D	Women with low grade abnormalities<br>(N=4201)	Diagnostic work up phase	0.01 (immediate treatment vs. biopsy & recall)<br>0.02 (cytological surveillance vs. biopsy &recall)	30 months (M)	0.034 QALYs loss found in immediate treatment vs. Biopsy &recall;<br>0.052 QALYs loss found in cytological surveillance vs. biopsy &recall	High
Tosteson ANA, 2014	-Controlled cohort study<br>-Effect of false positive screening mammograms' on QoL[16]<br>-USA	Breast cancer	-Mammograms	-EQ-5D<br>-Rating scale	Women<br>(N=1028)	Diagnostic work up phase<br>False positive	False positive vs negative group there is no disutility found (EQ-5D)<br>0.01-0.02 (Rating scale)	1year (M)	0.01-0.02 QALY loss (Rating scale)	High
Vasarainen H, 2013	-Randomized controlled study (screening arm vs	Prostate cancer	Screening: PSA	RAND-36	Men (N=2100)	Screening phase	Screening participant vs reference group	T1 (at invitation) till T5 (diagnostic	No QALY loss	Medium

Author Publication Year	Study Overview (a, Study design; b) Aim; c. Country)	Cancer Type	Intervention	Instrument used for utility measurement	Respondents (N=sample size)	Disutility Typology	Disutility Value Mean (95%CI if reported)	Time Frame （A=assumption, G=guideline, M=measurement）	QALY# or other findings	Quality Assessment Result
	control arm)<br>-Screening effect on QoL<br>-Finland		Diagnostic:<br>-DRE<br>-TRUS<br>-biopsy			Diagnostic work up phase	(age-stratified general male population) : no disutility found	examination by TRUS and biopsy) (detail not available)		
Whynes DK, 2008	-Randomized controlled study<br>-Alternative strategy on management of low grade abnormality<br>-UK	Cervical cancer	-colposcopy<br>-cytology	VAS	Women with borderline and low-grade abnormal smears (N=190)	Diagnostic work up phase	0.005-0.05 (surveillance vs. colposcopy)	30 months (M)	0.01- 0.025 QALY loss found in Surveillance group vs colposcopy	Medium

Notes: ASCUS=atypical squamous cells of undetermined significance; BCSP=Group Health's Breast Cancer screening Program; BMD=borderline or mildly dyskaryotic ; CBE=Clinical Breast Examination; CIN=cervical intraepithelial neoplasia grade; CT= Computed tomography; DCE= Discrete Choice Experiment; DRE=digital rectal examination; EQ-5D=EuroQoL 5 Dimension; FOBT= Fecal Occult Blood Test; FP= False positive; HPV=Human papillomavirus; HRQL=health related quality of life; HSIL=high--graded squamous intraepithelial lesion; HUI= Health Utilities Index; LBC=Liquid-based cytology; LSIL=low-graded squamous intraepithelial lesion; MM = mammography; MRI=Magnetic Resonance Imaging; PSA=prostate specific antigen; QALY#=Quality Adjusted Life Years (through calculation);SF-6D=Short Form 6 Dimension; SF-36=Short Form 36; SG=Standard Gamble; TN=True negative; TP=True positive; TRUS=trans-rectal ultrasound; TTO=Time Trade Off; VAS=Visual Analog Scale; US =Ultrasound; VIA=visual inspection with acetic acid.

##：The article references disutilities from the other related source; this article though is included instead because the original cited reference was not available.
