# Supplementary material for: Disutility associated with cancer screening programs: A systematic review
Source: PLoS One. 2019 Jul 24;14(7):e0220148. doi: 10.1371/journal.pone.0220148 (PMC6655768; doi:10.1371/journal.pone.0220148)
Supplement: S2 Table — (PDF) [file pone.0220148.s005.pdf]

Data extraction form of publications with estimated disutility values##

Author<br>Publication Year	Study Overview<br>(a) Study design; b) Aim;<br>(c) Country	Cancer Type	Screening modality	Instrument used for utility measurement	Disutility Typology	Disutility Value	Time Frame<br>（A=assumption, G=guideline, M=measurement）	QALY (Quality Adjusted Life Year) loss or other findings
Berkhof J, 2010	(a) Modeling study<br>(b) -Assess cost-effectiveness of different screening strategies<br>(c) The Netherlands	Cervical cancer	-cytology test<br>-HPV test	Estimation	Diagnostic work up phase<br>Positive test result	0.03	1 month (A)	0.0025 QALY loss
					CIN1	0.03	6 months (A)	0.015 QALY loss
					CIN2	0.07	6 months (A)	0.035 QALY loss
					CIN3	0.07	6 months (A)	0.035 QALY loss
De Bekker-Grob EW, 2012	(a) Randomized controlled trial<br>(b) Compare cost-effectiveness of LBC vs. CP<br>(c) The Netherlands	Cervical cancer	Screening:<br>-LBC<br>-CP<br>-HPV Diagnostic:<br>-Colposcopy	Estimation	Screening phase<br>CP<br>LBC<br>HPV	0.006	2 weeks (A)	0.000231 QALY loss
					Diagnostic work up phase<br>False Positive	0.03	1 month (A)	0.0025 QALY loss
					CIN1[4]	0.03	6 months (A)	0.015 QALY loss
					CIN2	0.07	1 year (A)	0.07 QALY loss
					CIN3	0.07	1 year (A)	0.07 QALY loss
Goede SL, 2017	(a) Modeling study<br>(b) Estimate the benefit-harms and cost of FIT vs. FOBT<br>(c) Canada	Colorectal cancer	-FIT<br>-FOBT<br>Diagnostic:<br>-colonoscopy	Estimation	Diagnostic work up phase<br>Colonoscopy	1	2 days (A)	0.0055 QALY loss
					Complications(bleeding, perforation)	1	2 weeks (A)	0.0384 QALY loss
Kitchener HC, 2011	(a) Randomized Control study<br>(b) Compare automation-assisted reading vs. manually reading in cytology<br>(c) UK	Cervical cancer	-Cytology<br>Diagnostic:<br>-HPV<br>-colposcopy	Estimation (based on HUI** & TTO*)	Diagnostic work up phase<br>false positive	0.04	6 months (A)	0.02 QALY loss
					CIN1	0.11	6 months (A)	0.055 QALY loss
					CIN2	0.12	6 months (A)	0.06 QALY loss
					CIN3	0.11	6 months (A)	0.055 QALY loss
Manser R, 2005	(a) Modeling study<br>(b) Assess the cost effectiveness of screening with low dose spiral CT<br>(c) Australia	Lung cancer	Low dose spiral CT	Estimation	Diagnostic work up phase<br>False positive	0.02	6 months until next follow up(A)	0.01 QALY loss
					Treatment phase<br>Overtreatment	0.12	Until death from other causes(A)	

Author<br>Publication Year	Study Overview<br>(a) Study design; b) Aim;<br>(c) Country	Cancer Type	Screening modality	Instrument used for utility measurement	Disutility Typology	Disutility Value	Time Frame<br>（A=assumption, G=guideline, M=measurement）	QALY (Quality Adjusted Life Year) loss or other findings
Naber SK, 2018	(a) Modeling study<br>(b) Evaluate the cost-effectiveness of screening for people with family history of colorectal cancer<br>(c) USA	Colorectal cancer	Colonoscopy	Estimation	Screening phase<br>Colonoscopy	0.5	1.5 days (A)	0.0020 QALY loss
					Complication (colonoscopy)	0.5	2-4 days (A)	0.0027-0.0055 QALY loss
Pataky R, 2014	(a) Modeling study<br>(b) Assess the cost effectiveness of different screening strategies<br>(c) Canada	Breast cancer	Mammography<br>Diagnostic:<br>Biopsy	Estimation	Screening phase	No disutility reported	Not available	
					Diagnostic work up phase<br>Diagnostic mammography	0.158	2 weeks (A)	0.0061 QALY loss
Raab SS, 1997	(a) Modeling study<br>(b) Assess risk-taking attitude's effect on cost effectiveness of testing strategies<br>(c) USA	Lung cancer	-Sputum<br>-Fine-needle aspiration<br>- Bronchoscopy<br>-Thoracoscopy	Estimation	Diagnostic work up phase<br>False positive	0.2<br>0.6	1month (A)<br>4months (A)	0.017-0.2 QALY loss
					Indeterminate diagnosis	0.2	1 month (A)	0.017 QALY loss
					Sputa test	0.1	1 week (A)	0.0019 QALY loss
					Fine needle aspiration	0.1	4 days (A)	0.0011 QALY loss
					Thoracoscopy	0.3<br>0.5	1 month (A)<br>4 months (A)	0.025-0.1667 QALY loss
Stout NK, 2006	(a) Modeling study<br>(b) Evaluate the cost-effectiveness mammography screening<br>(c) USA	Breast cancer	-Mammography	Estimation	Screening phase	0.75	7 days (A)	0.0144 QALY loss
					Diagnostic work up phase<br>False positive	075	25 days (A)	0.0514 QALY loss
Van Rosmalen J, 2012	(a) Modeling study<br>(b) Assess the cost effectiveness of cytology vs. HPV DNA testing<br>(c) The Netherlands	Cervical cancer	-cytology<br>-HPV	Estimation	Screening phase<br>Pap smear<br>HPV test	0.006	2 weeks (A)	0.00023 QALY loss
					Diagnostic work up phase<br>False positive	0.005	6 month (A)	0.0025 QALY loss
					CIN1	0.03	6 month (A)	0.015 QALY loss

Author<br>Publication Year	Study Overview<br>(a) Study design; b) Aim;<br>(c) Country	Cancer Type	Screening modality	Instrument used for utility measurement	Disutility Typology	Disutility Value	Time Frame<br>（A=assumption,<br>G=guideline,<br>M=measurement）	QALY (Quality Adjusted Life Year) loss<br>or other findings
					CIN2	0.07	1 year (A)	0.07 QALY loss
					CIN3	0.07	1 year (A)	0.07 QALY loss
					Diagnostic work up phase<br>Immediate HPV	0.0033	18 months (G)	
Van Hees F, 2014	(a) Modeling study<br>(b) Evaluate the screening strategy for elderly aged over 75 without prior screening<br>(c) The Netherlands	Colorectal cancer	-FIT<br>-Sigmoidoscopy<br>-Colonoscopy	Estimation	Screening phase<br>FIT	No utility loss		
					Sigmoidocscopy	1	1 day (A)	0.0027 QALY loss
					Colonoscopy<br>Complication (colono scopy)	1<br>1	2 days (A)<br>2 weeks (A)	0.0055 QALY loss<br>0.0384 QALY loss
					Treatment phase<br>Overtreatment	0.05-0.70 per life year	Until death of other cause (A)	0.05-0.70 QALY loss (depends diagnosed cancer stage, from stage1 – 4)

Notes: CIN=cervical intraepithelial neoplasia grade; CP=Conventional Papanicolaou smear; CT= Computed tomography; DRE=digital rectal examination; FIT=Fecal Immunofluorenscence Test；FOBT=Fecal Occult Blood Test; HPV=Human papillomavirus; LBC=Liquid-based cytology; PSA=prostate specific antigen; TRUS=trans-rectal ultrasound,

*the value adjusted based on TTO measurement from Myers ER; ** the value adjusted based on HUI measurement from Stratton KR

##：If author used disutility from the literature but also applied certain assumptions for the value, and therefore its value differ from the cited value, then it was considered as estimated value.
