# Supplementary material for: Disutility associated with cancer screening programs: A systematic review
Source: PLoS One. 2019 Jul 24;14(7):e0220148. doi: 10.1371/journal.pone.0220148 (PMC6655768; doi:10.1371/journal.pone.0220148)
Supplement: S3 Table — (PDF) [file pone.0220148.s006.pdf]

Summary Table of Quality Appraisal

Studies	Relevance to the population's preference	Quality assessment—Risk of bias	Appropriateness of measure of disutility values	Time frame	General Grading : High, Medium, Low, Very Low
Bonomi AE 2008	Fair	poor	Good	Fair	Low
Drolet M 2011	Good	Good	Good	Good	High
Birch S 2002	Fair	Poor	Good	Fair	Low
Essink-Bot ML 1998	Fair	Good	Good	Good	High
Haes JCJM 1991	Poor	Poor	Good	Fair	Low
Gyrd-Hansen D 2001	Good	Good	Good	NA	High
Insinga RP 2007/Myers ER 2004	Fair	Poor	Good	Good	Medium
Stratton KR 2000	Poor	Poor	Good	Fair	Low
De Kok IMCM 2018	Good	Good	Good	Fair	High
Koning HJ 1991	Poor	Poor	Good	Poor	Very Low
Maissi E 2005	Fair	Good	Good	Good	High
Mo X 2017	Fair	Poor	Fair	Poor	Very Low
Tosteson ANA 2014	Good	Good	Good	Good	High
Vasarainen H 2013	Good	Good	Good	Poor	Medium
Howard K 2008	Good	Fair	Good	Fair	Medium
Cormier L 2002	Good	Fair	Good	Good	High
Gerard K 1999	Good	Fair	Good	Poor	Medium
Howard K 2009	Fair	Good	Good	NA	Medium
Johnston K 1998	Good	Fair	Good	Fair	Medium
Marshall DA 2009	Good	Good	Good	NA	High
Melnikow J 2002	Fair	Poor	Good	Poor	Low
Rijnsburger AJ 2004	Good	Good	Good	Good	High

Simonella L 2014	Fair	Poor	Good	Poor	Low
Whynes DK 2008	Fair	Fair	Good	Good	Medium
Cantor SB2008	Fair	Fair	Good	Good	Medium
TOMBOLA group 2009	Good	Good	Good	Good	High
Ock M 2016	Fair	Good	Good	Poor	Medium
